# Evaluation of Corticospinal and Intracortical Excitability After Stroke Using Paired-Pulse Transcranial Magnetic Stimulation: A Narrative Review

**DOI:** 10.3390/life16071148

**Published:** 2026-07-11

**Authors:** Maria Caputo, Davide Giannuzzi, Stefano Bonomi, Chiara Piccininni, Cristina Cuccagna, Augusto Fusco

**Affiliations:** 1 Department of Neurology, Carpi Hospital, 41012 Modena, Italy; m.caputo@ausl.mo.it; 2 Department of Biomedical, Metabolic and Neural Sciences, University of Modena and Reggio Emilia, 41125 Modena, Italy; 3 UOSD High-Intensity Neurorehabilitation, Department of Neuroscience, Sensory Organs and Thorax, University Hospital Foundation “Agostino Gemelli” IRCCS, 00168 Rome, Italy; davide.giannuzzi@guest.policlinicogemelli.it (D.G.); chiara.piccininni@policlinicogemelli.it (C.P.); cristina.cuccagna@policlinicogemelli.it (C.C.); augusto.fusco@policlinicogemelli.it (A.F.)

**Keywords:** stroke, neural plasticity, rTMS, ppTMS, neurorehabilitation

## Abstract

**Background**: Alterations in corticospinal and intracortical excitability are frequently observed following a stroke. Cortical plasticity and reorganization after stroke can be assessed using transcranial magnetic stimulation (TMS) measures. These include Motor Threshold (MT) and Motor Evoked Potential (MEP) obtained from single-pulse TMS and Short-Interval Intracortical inhibition (SICI) and Intracortical Facilitation (ICF) obtained from Paired-Pulse Paradigms (ppTMS). **Objective**: This narrative review aims to provide an update on the most relevant studies and clinically interpret alterations in excitability measures from ppTMS studies following a stroke, focusing on the effects of stroke on MT, MEP, SICI, and ICF in both hemispheres. Additionally, we analyze intracortical disinhibition across different stages of recovery and the implications for inter-hemispheric competition during recovery. **Literature search strategy**: Studies investigating corticospinal excitability and intracortical inhibition/facilitation after stroke using ppTMS paradigms were reviewed. The main neurophysiological outcomes included MT, MEP amplitude, SICI, and ICF, comparing the affected and unaffected hemispheres and healthy controls. **Results**: The reviewed literature consistently demonstrates reduced corticospinal excitability in the affected hemisphere, characterized by an increased MT and decreased MEP amplitude. Conversely, intracortical inhibitory and facilitatory circuits appear to be altered after stroke. Reduced SICI and modified ICF patterns suggest cortical disinhibition. **Conclusions**: Assessments based on ppTMS can be considered significant indicators of post-stroke cortical reorganization. Cortical motor inhibition may have an adaptable or stage-specific role, potentially beneficial in the acute and subacute stages, but harmful if it continues beyond the chronic phase.

## 1. Introduction

Stroke remains the second leading cause of death and the third leading cause of death and disability combined (as expressed by disability-adjusted life-years lost) worldwide [1]. The neurophysiological mechanisms underlying functional recovery or persistent neurological deficits that underpin functional recovery or persistent neurological deficits following a stroke remain incompletely understood. Alterations in corticospinal [2] and intracortical [3] excitability are recognized as key neurophysiological features of stroke. These changes reflect the complex processes of neural reorganization and plasticity that emerge after brain injury [4].

During the acute phase, partial improvements in impairments may result from the resolution of perilesional edema and/or diaschisis, which can impact regions distant from the lesion site [5], as well as from various environmental and physical stimuli [6]. In contrast, post-acute recovery is primarily attributed to activity-dependent brain plasticity [6].

A comprehensive understanding of the effects on corticospinal excitability may facilitate the development of targeted interventions aimed at enhancing the functional recovery in individuals with stroke [4]. Previous studies have mainly focused on molecular and physiological processes involved in cortical plasticity, whereas a limited number have examined mechanisms within the cortex. Experimental studies suggest that the occurrence of adaptive plastic mechanisms in the primary motor cortex (M1) needs a reduction in local inhibitory circuitry. This local disinhibition is believed to uncover existing but hidden corticocortical connections, thereby promoting structural and functional plasticity and recovery [7]. Changes in cortical organization are also linked to various mechanisms, including revealing pre-existing but inactive corticocortical connections, changing synaptic strength through long-term potentiation (LTP) and long-term depression (LTD), and forming new synaptic connections [7].

Transcranial magnetic stimulation (TMS) allows the study of both short- and long-term patterns of motor cortex reorganization during motor recovery after stroke. Paired-pulse TMS (ppTMS) paradigms are considered the best method for assessing the excitability of inhibitory and excitatory intracortical circuits in the human motor cortex and evaluating how these circuits contribute to modulating corticospinal output [8].

Recently, Washabaugh and colleagues [4] have identified quantitative changes in TMS-derived measures associated with stroke but did not provide a clinical interpretation of these changes relative to the time course of recovery from stroke. In healthy individuals, interhemispheric differences in TMS-derived measures are minimal, relatively stable, and only marginally influenced by experimental conditions [4]. Despite the growing number of studies using transcranial magnetic stimulation in people with stroke, TMS-derived neurophysiological findings remain heterogeneous and only partly integrated with clinical outcomes, limiting their routine translation into neurorehabilitation practice [9]. Recent systematic reviews and overviews of non-invasive brain stimulation in stroke rehabilitation report mixed results with modest effect sizes, considerable variability in stimulation protocols, timing, and patient selection, and overall low-to-moderate certainty of evidence. Consequently, TMS and other NIBS techniques are still not recommended as standard of care, both as diagnostic and therapeutic interventions, in most clinical guidelines [10,11].

This narrative review aims to address the following three questions. First, it examines how corticospinal and intracortical excitability are altered by stroke in both hemispheres. This analysis involves the assessment of Motor Threshold (MT) and Motor Evoked Potential (MEP) obtained from single-pulse TMS and Short-Interval Intracortical inhibition (SICI) and Intracortical Facilitation (ICF) obtained from Paired-Pulse Paradigms. MT and MEP are indicators of corticospinal excitability, with MT representing the minimal magnetic stimulation required to activate the motor cortex and produce a muscle response, and MEP indicating the magnitude of the muscle response generated at a specific stimulation intensity [2]. On the other hand, SICI and ICF assess intracortical excitability, as SICI reflects the degree of inhibitory activity within the motor cortex, and ICF reflects the level of facilitatory (excitatory) activity within intracortical neural circuits [3].

Second, this review delineates the temporal progression of intracortical disinhibition as patients recover from stroke during the acute, subacute, and chronic phases of their recovery (during the first few weeks and up to 6 months).

Third, the findings of this review aim to provide insights into the inter-hemisphere competition hypothesis, evaluating the applicability of the non-invasive neuromodulation in stage-specific recovery.

## 2. Materials and Methods

### 2.1. Neurophysiological Transcranial Magnetic Stimulation Parameters

The study of neurophysiological parameters through TMS enables non-invasive assessment of motor system physiology after stroke. Evidence suggests that TMS may aid in predicting motor recovery and stratifying patients in post-stroke clinical trials [12]. TMS coils are applied over the motor cortex relative to the side of the brain injury. Neuropharmacological TMS studies have demonstrated that a suprathreshold test stimulus delivered after a subthreshold conditioning stimulus delivered close together at very short inter-stimulus intervals (ISI) activates gamma-aminobutyric acid type A (GABA_A) interneuronal activity and produces intracortical inhibition (ICI), resulting in the suppression of motor evoked potentials (MEP) [13]. Conversely, facilitation observed at longer ISIs with two maximal conditioning and test stimuli is believed to reflect the activation of glutamatergic interneurons mediating intracortical facilitation (ICF) [14]. Accumulating evidence indicates that ICI and ICF, assessed using ppTMS paradigms, represent the activity of distinct inhibitory and excitatory interneuronal circuits within the motor cortex, respectively. Furthermore, inhibitory interneurons appear to have lower activation thresholds than excitatory interneurons [15]. The relationship between alterations in ICI/ICF recovery curves and motor cortex plasticity has been investigated under both physiological and pathological conditions. In individuals with stroke, ppTMS studies have revealed that changes in intracortical excitability in both the affected hemisphere (AH) and the unaffected hemisphere (UH) may represent neurophysiological markers associated with motor recovery [4,6].

### 2.2. Literature Search Strategy

A narrative review of the literature was conducted to evaluate post-stroke changes in corticospinal and intracortical excitability assessed using transcranial magnetic stimulation (TMS). This review focused on the main neurophysiological measures used in stroke studies, including motor threshold, motor evoked potential amplitude, short-interval intracortical inhibition, and intracortical facilitation, with comparisons across the affected hemisphere, unaffected hemisphere, and healthy control participants. In this study, we aim to provide a focused synthesis of key studies relevant to the research question, establishing the theoretical and empirical background for the current investigation. Consequently, a narrative review was considered more appropriate than a systematic or scoping review. A comprehensive search of relevant articles was conducted using the PubMed database, including studies published until the end of 2025. Studies were screened based on title and abstract, followed by full-text evaluation when necessary. The keywords used for the literature search included “Cortical Excitability”, “Paired-Pulse TMS (ppTMS)”, “Repetitive TMS (rTMS)”, “Stroke”, “Non-Invasive Brain Stimulation”, and “Neurorehabilitation”. TMS measurements were compared between the affected and unaffected hemispheres of individuals with stroke.

To be included in this review, studies were required to be published in English, present original research involving individuals who had experienced a stroke, and employ either cross-sectional or prospective case–control designs examining corticospinal or intracortical excitability following stroke using single-pulse or paired-pulse transcranial magnetic stimulation (TMS) protocols, while reporting neurophysiological measures related to motor cortex function. In addition, meta-analyses meeting the same clinical and methodological criteria and focusing on TMS-derived parameters could be included as a higher-level quantitative synthesis to contextualize and strengthen the narrative interpretation of individual study findings.

Studies investigating other neurological or medical conditions and those that did not meet the predefined inclusion criteria were excluded.

## 3. Results

A total of 11 studies were included: meta-analysis (1), case–control studies (9), and a retrospective study (1) [3,4,6,16,17,18,19,20,21,22,23]. Most of these studies reported a reduction in cortical excitability in the AH post-stroke, through an increase in motor threshold (MT) and decreased amplitude of MEPs compared to both UH and control subjects. In contrast, SICI levels were reduced in the AH group, suggesting an intracortical disinhibition state. Furthermore, bilateral reductions in intracortical inhibition have been observed after stroke, with more pronounced changes in the AH (see Table 1).

The ICF results varied across studies. Nonetheless, several studies reported an increase in ICF in the AH compared with the UH, whereas the UH shows less facilitation than expected in control subjects [4,6].

**Figure 1:**
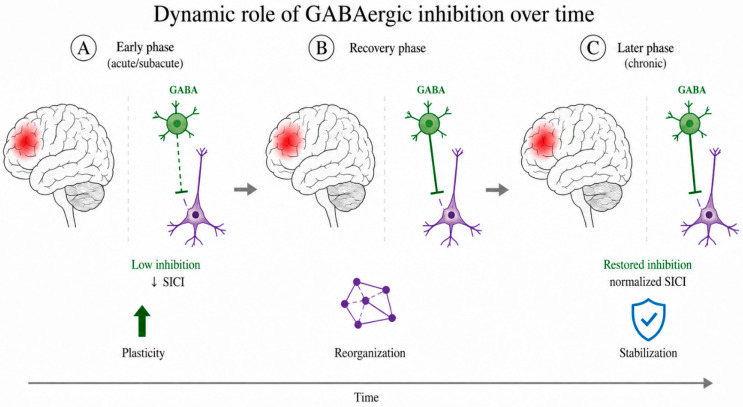
Dynamic role of GABAergic inhibition during post-stroke motor recovery. (**A**) In the early acute/subacute phase, reduced GABAergic inhibition and decreased SICI may facilitate cortical plasticity and the unmasking of latent motor pathways. (**B**) During the recovery phase, progressive reorganization of motor networks occurs, supporting functional improvement. (**C**) In the later chronic phase, restoration of inhibitory control and normalization of SICI are likely important for stabilizing newly formed motor representations and improving the efficiency of motor functions.

Longitudinal data revealed partial recovery of corticospinal excitability over time for both the mean amplitude of the MT and progressive normalization of the MEP. Additionally, SICI exhibited partial recovery during follow-up, particularly in younger patients (Figure 1) (Table 1) [4,24].

## 4. Discussion

### 4.1. Mechanisms of Plasticity After Stroke

Ischemic stroke induces a range of neurophysiological responses involving excitatory and inhibitory intracortical circuits within M1. These changes are associated with the level of motor impairment and the potential for functional recovery [4]. Consistent findings have shown a significant decrease in corticospinal excitability in AH, as reflected by increased MT and reduced MEP amplitudes [4,16,21]. A progressive slight improvement has been demonstrated over the months, with a progressive increase in MEP amplitude and a decrease in MT from the first few weeks after stroke up to the following 6 months [4]. This reduction is predominantly observed in the AH, while the UH resembles that of healthy controls [25] (see Table 2).

The central impairment may also lead to failure to increase cortical excitability in response to increased cortical inhibition during muscle contraction, which may be linked to the high physical fatigue reported by stroke patients. It has been shown that physical fatigue in stroke is also associated with prolonged cortical silent period durations and reduced cortical voluntary activation, particularly in the AH [26]. The equilibrium between intracortical inhibition and facilitation is a critical factor in determining motor output and cortical network adaptation after injury [22]. SICI, mainly mediated through GABAergic interneuronal circuits, has been identified as an important indicator of inhibitory circuit function in the M1 area [3]. Physiologically, SICI suppresses neuronal populations that may interfere with the generation of the desired motor command or cause unnecessary co-contraction of muscles. After a stroke, TMS studies have shown reductions in SICI in both AH and UH during the acute and subacute phases [19,20]. This reduction reflects intracortical disinhibition and suggests a disruption of GABAergic inhibitory networks. Mechanistically, the defective intracortical inhibition observed in the AH may reflect either a persistent functional impairment of the GABAergic inhibitory circuits within M1 or an increased excitability and a lowered activation threshold of the interneurons responsible for facilitation at short interstimulus intervals. Currently, the ppTMS data do not allow for a conclusive distinction.

**Figure 2:**
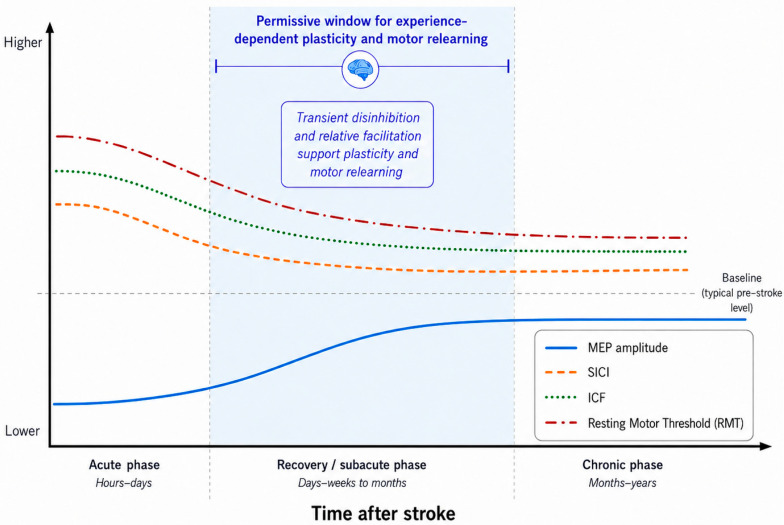
Longitudinal changes in motor cortical excitability after stroke.

Limited evidence is available on how SICI changes during the chronic phase after stroke. However, existing studies suggest that sustained intracortical disinhibition is linked to poor motor outcomes, including decreased dexterity, reduced motor performance, and impaired motor control [16]. Takechi and colleagues [16] reported that SICI in AH remained reduced for up to one year, whereas SICI in the UH returned to normal values immediately following intensive rehabilitation (Table 2, Figure 2). Individuals who achieved good recovery exhibited normalization of reduced SICI in the UH within three months, while those with poor recovery continued to exhibit abnormal SICI levels [19]. These findings indicate that decreased SICI in the UH may represent an early adaptive response to brain injury, promoting functional reorganization and recovery. A reduction in SICI may contribute to enhancing motor output from the AH, also showing neuroplastic changes and functional reorganization across both hemispheres.

This apparent paradox suggests that disinhibition may be beneficial only within a specific temporal window, beyond which it may reflect a maladaptive network reorganization. Specifically, an excessive reduction in inhibitory control may lead to inefficient motor output, increased variability, and impaired task-specific motor learning [3].

Concurrently, ICF, primarily mediated by glutamatergic (N-methyl-D-aspartate (NMDA)-dependent) circuits, shows more heterogeneous changes. While some studies report increased facilitation in the AH, others observe preserved ICF depending on stroke severity, lesion location, and time since injury [4,22] (Figure 2). This variability highlights the dynamic and context-dependent nature of excitatory intracortical processes during the recovery period. The observed disinhibition and facilitation could be compensatory mechanisms that account for reduced corticospinal excitability after stroke [4].

The relative preservation of ICF may indicate that excitatory intracortical circuits are less vulnerable to ischemic damage or recover more rapidly than inhibitory networks. Alternatively, this may reflect the methodological limitations in detecting subtle changes in the facilitatory pathways using current TMS paradigms.

A key concept emerging from the literature is that post-stroke neuroplasticity is highly dependent on the disease stage. This temporal framing is grounded in the longitudinal and phase-specific studies included in the present review: intracortical disinhibition is detectable in the acute phase [20] and follows a measurable trajectory as recovery progresses [16]. In the acute and subacute phases, reduced inhibition may facilitate cortical reorganization and functional brain recovery after stroke. In contrast, during the chronic phase, persistent disinhibition may become maladaptive and contribute to impaired motor control [3].

### 4.2. Interhemispheric Imbalance and Motor Recovery

A critical framework for interpreting post-stroke neurophysiology is the interhemispheric competition model [27].

**Figure 3:**
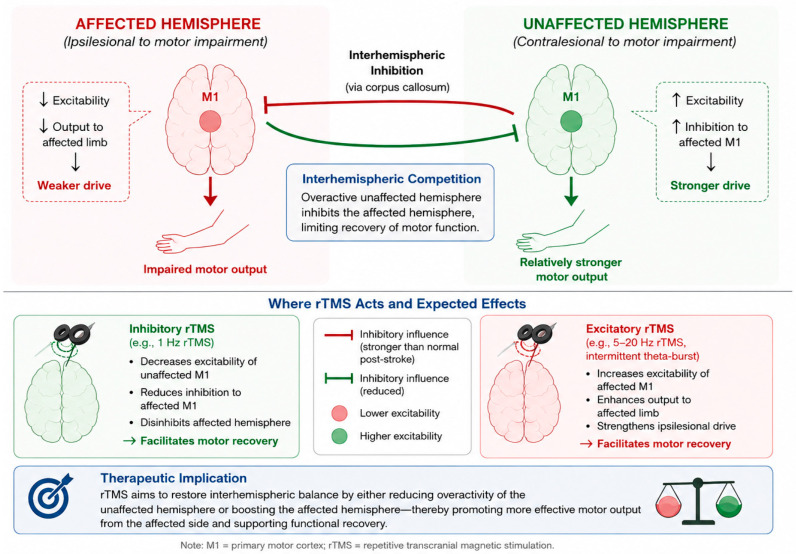
Interhemispheric model of post-stroke motor dysfunction and imbalance. Expected effects of rTMS in the modulation of cortical excitability. “↑” indicates increases whereas “↓“ decreases in the measured parameter.

According to the aforementioned hypothesis, the hemispheres are functionally connected through transcallosal pathways, thereby maintaining equilibrium via mutual inhibition. However, this balance is disrupted following brain injury. The reciprocal inhibitory modulation between the hemispheres becomes asymmetrical, with the uninjured hemisphere exerting excessive pathological inhibition on the damaged hemisphere, further reducing its functionality [28] (Figure 3). An increase in inhibition in the intact hemisphere after mild-to-moderate stroke occurs simultaneously with decreased inhibitory activity within the infarcted hemisphere due to changes in GABAergic regulation. This creates a difference in inhibitory control between the two cerebral hemispheres [19]. The involved mechanisms include disruption of transcallosal inhibition and ipsilateral silent period (iSP) abnormalities, both of which indicate cortical disinhibition [29]. The reduction in motor inhibition in the AH can expose previously inhibited pathways, thus allowing the contralateral hemisphere to influence function through the activation of corticocortical pathways [30]. However, excessive inhibition may hinder cortical reorganization around the damage site, thereby limiting motor recovery post-stroke [31].

This model is supported by evidence of altered interhemispheric inhibition (IHI) following stroke, as measured using the iSP and ppTMS paradigms. Nevertheless, the functional significance of this imbalance remains unclear. Some studies have associated increased contralesional excitability with poorer motor outcomes, while others have shown that contralesional activation may support compensatory mechanisms, particularly in patients with extensive corticospinal tract damage [32].

Grefkes proposed that alterations in motor system activity are closely linked to functional recovery after stroke [9]. The connectivity pattern between the two primary motor cortices (M1) changes over time and is influenced by stroke severity. During the acute stage, the inhibitory influence of the ipsilesional M1 on the contralesional M1 is significantly reduced, especially in individuals with severe impairments, as demonstrated by neurophysiological findings showing decreased SICI [16]. Approximately two weeks following a stroke, the contralesional M1 starts to facilitate activity in the ipsilesional M1, suggesting a compensatory function in aiding motor function during the subacute phase. The facilitatory influence is reflected in the measures of ICF [4]. As recovery progresses over months, the ipsilesional motor cortex gradually restores its inhibitory control over the contralesional M1. In subjects with favorable motor recovery, the contralesional M1 may continue to provide beneficial support for activities in the AH. On the other hand, individuals with poorer functional outcomes often lose supportive interactions [9].

The shift in the contralesional M1 from a facilitatory to an inhibitory influence is considered a maladaptive form of neuroplasticity that may hinder motor recovery and contribute to persistent deficits in the paretic limb. Although the mechanisms underlying this maladaptive reorganization remain unclear, lesion location appears to be a contributing factor [9,33]. Evidence from longitudinal studies suggests that inter-hemisphere imbalances are dynamic rather than fixed and evolve over time. In the initial phase after stroke, decreased inhibitory output from the AH may support reorganization and recovery. Over time, improvements in inter-hemisphere equilibrium and functional improvements have been found to be partially restored, although this process is highly variable among individuals [4].

These results highlight the dual role of GABAergic circuits in the process of motor recovery. Initially, GABAergic inhibition may serve to limit plasticity but later becomes important for stabilizing new motor representation. Therefore, the relationship between SICI and motor function is not linear but is dynamically regulated by time.

### 4.3. Therapeutic Implications and Neuromodulation Strategies

Non-invasive brain stimulation (NIBS) has been investigated for its capacity to modulate motor recovery after stroke and influence motor, sensory, and cognitive functions [33]. Different studies have suggested that brain stimulation may enhance the beneficial effects of motor training during rehabilitation by targeting specific functional neural circuits and modulating cortical excitability and movement-related activation [34].

**Figure 4:**
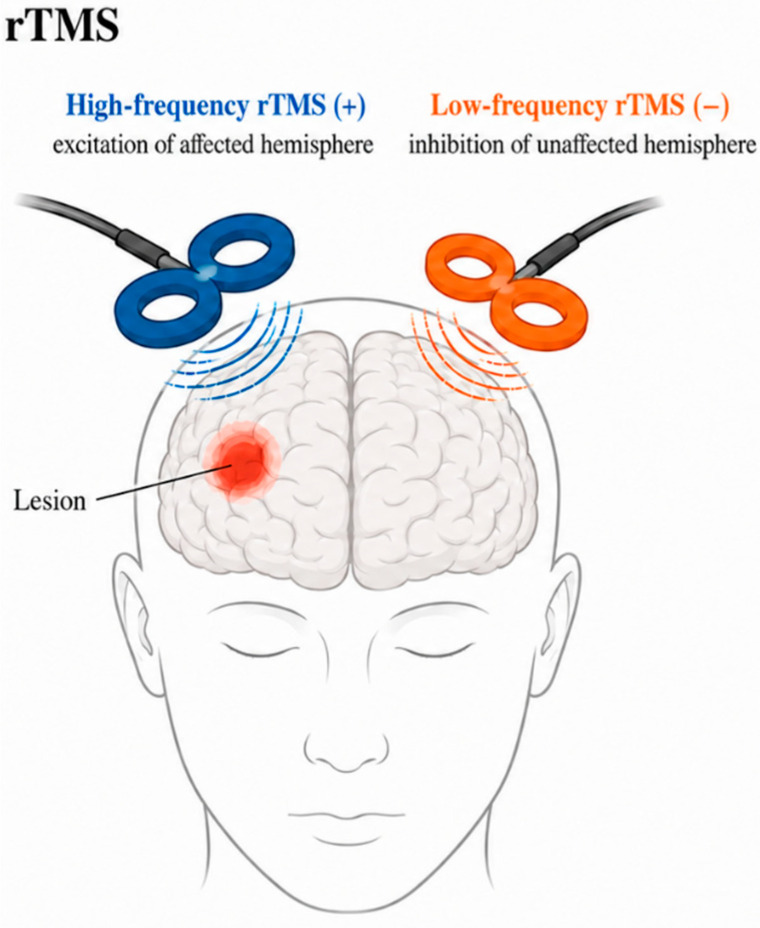
Diagram of the high-frequency rTMS over the lesioned hemisphere is generally excitatory, whereas low-frequency rTMS over the unaffected hemisphere is inhibitory.

Traditional approaches, grounded in the interhemispheric competition model, aim to restore hemispheric balance by either increasing excitability in the ipsilesional motor cortex or suppressing activity in the contralesional hemisphere using NIBS techniques, such as repetitive TMS (rTMS) (Figure 4) and tDCS. However, the clinical translation of this principle is still inconsistent. A multicenter randomized trial of contralesional low-frequency rTMS (the NICHE trial) found no benefit over sham when added to motor training [35], and evidence-based guidelines accordingly assign only limited recommendations for rTMS in post-stroke motor recovery [36].

rTMS can modulate cortical excitability, and when delivered as theta-burst stimulation (TBS), it can induce LTP- or LTD-like effects on the brain. Motor and behavioral gains from rTMS protocols may be maximized when brain stimulation is coupled with carefully designed rehabilitation [37]. Di Lazzaro and colleagues [38] investigated the effects of TBS on cortical excitability in acute stroke patients and demonstrated that both facilitatory TBS applied over the affected motor cortex and inhibitory TBS applied over the unaffected hemisphere significantly increased the amplitude of MEPs elicited from the lesioned hemisphere. These effects were comparable to those observed in healthy controls, suggesting that facilitatory stimulation of the affected hemisphere and inhibitory stimulation of the intact hemisphere enhance the excitability of the lesioned motor cortex during the acute phase of stroke recovery.

Despite the strong theoretical rationale supporting these interventions, their clinical outcomes have remained inconsistent. While some studies have reported improvements in motor function following cortical excitability modulation, others have failed to demonstrate significant or long-lasting benefits [38]. For example, patients with reduced ipsilesional M1 activity, as measured by fMRI, experienced worsening motor performance after excitatory stimulation of the ipsilesional M1, suggesting that cortical modulation may not be universally beneficial [39]. Conversely, inhibitory rTMS applied to the contralesional M1 proved effective in patients exhibiting hyperactivity of the contralesional precentral gyrus during movements of the paretic hand [40]. Furthermore, connectivity analyses revealed that the greatest benefits from contralesional inhibitory rTMS were observed in patients whose intervention successfully reduced abnormally increased interhemispheric inhibition between the primary motor cortices [39]. These findings highlight that the effectiveness of non-invasive brain stimulation may depend on individual patterns of cortical activity and connectivity, underscoring the need for more personalized therapeutic approaches.

Such variability likely reflects the complexity of post-stroke neuroplasticity and the limitations of interpreting recovery solely through an interhemispheric competition framework [9]. Increasing evidence suggests that successful motor recovery depends not only on the modulation of cortical excitability but also on the restoration of appropriate intracortical inhibitory mechanisms [41].

The gap between the mechanistic rationale and clinical outcomes motivates the stage-specific interpretation of excitability data adopted in this review. Therapeutic interventions should be tailored to the time course of recovery rather than applied uniformly across all stages of the recovery.

### 4.4. Limitations and Future Directions

Despite substantial progress in understanding post-stroke cortical excitability, several limitations still remain. First, the variability in TMS methodologies across studies complicates direct comparisons. Differences in stimulation parameters, ISIs, and patient characteristics may have contributed to these inconsistent findings.

Second, most studies rely on cross-sectional designs, limiting the ability to infer causal relationships between neurophysiological changes and functional recovery in people with stroke. Longitudinal studies are needed to clarify the temporal dynamics of intracortical inhibition and facilitation.

Third, current TMS measures provide only indirect indices of cortical circuit function and do not capture the full complexity of large-scale network interaction. Integration with neuroimaging techniques, such as functional magnetic resonance imaging and diffusion tensor imaging, may provide a more comprehensive understanding of post-stroke brain reorganization.

Fourth, the included studies were selected using a non-systematic literature search and did not undergo formal quality appraisal (risk of bias) to evaluate their methodological quality. The lack of a structured risk-of-bias assessment using standardized tools (e.g., domain-based instruments developed for randomized and non-randomized studies) limits the strength of the results of the narrative reviews. In accordance with this concern, we did not perform a new quantitative synthesis because of the substantial methodological heterogeneity across the included studies (differences in stimulation parameters, inter-stimulus intervals, and patient characteristics), which precluded meaningful pooling of effect sizes in a narrative format. Lesion location and type were not consistently reported across the included studies, which prevented systematic stratifications of the neurophysiological findings. In this study, we aimed to report qualitative findings that should be interpreted as complementary to the quantitative findings from the systematic meta-analysis results.

Further studies are required to determine whether TMS-induced electrophysiological changes translate into clinically meaningful improvements. In particular, large, controlled, longitudinal, and stage-specific studies are needed. As a result, we may determine whether different stimulation protocols match the phase of recovery and verify the role of intracortical excitability measures as biomarkers of stable functional recovery.

## 5. Conclusions

Recovery of post-stroke motor function involves changes in both the excitability of the corticospinal systems as well as within the cortex itself. The current literature suggests a model in which reduced corticospinal output coexists with intracortical disinhibition, with each showing distinct temporal patterns and functional implications. In addition, the extent of motor recovery does not appear to be dependent solely on an inter-hemispheric imbalance but rather on a cooperative organization and subsequent reorganization of excitatory/inhibitory circuits across the distributed motor network.

These findings underscore the importance of individualized, stage-specific rehabilitation strategies that aim to restore functional network balance rather than merely increasing or decreasing cortical excitability in one direction.

## Figures and Tables

**Table 1:** Identification and selection of studies included in the literature review, along with the ppTMS and TMS parameters analyzed. MT, motor threshold; MEP, motor evoked potential; SICI, short-interval intracortical inhibition; ICF, intracortical facilitation. In the parameter columns, ‘+’ indicates an increase and ‘−’ a decrease in the affected hemisphere relative to the unaffected hemisphere or healthy controls; NA indicates the parameter was not assessed.

Author, Year	Study Type	Sample Size	MT	MEP	SICI	ICF
Washabaugh et al., 2024 [4]	Meta-analysis	625	+	−	−	+
Takechi et al., 2014 [16]	Case–control	24	+	NA	−	NA
Hummel et al., 2009 [17]	Case–control	14	NA	NA	−	NA
Marques et al., 2025 [18]	Case–control	147	NA	NA	−	NA
Ding et al., 2019 [3]	Case–control	12	NA	NA	−	NA
Manganotti et al., 2002 [19]	Case–control	15	+	NA	−	NA
Liepert et al., 2000 [20]	Case–control	6	NA	NA	−	NA
Traversa et al., 1998 [21]	Case–control	17	NA	−	NA	NA
Cicinelli et al., 2003 [22]	Case–control	10	+	NA	−	NA
Shimizu et al., 2002 [23]	Case–control	12	NA	NA	−	NA
Seo et al., 2018 [6]	Retrospective	103	NA	NA	−	NA

**Table 2:** Summary of neurophysiological changes across the stages of post-stroke recovery. The table presents the typical direction of change in key neurophysiological measures relative to healthy controls during the acute and chronic phases after stroke. “+” indicates increases whereas “−” decreases in the measured parameter.

	Acute/Subacute		Chronic		References
	AH	UH	AH	UH	
SICI	−	−	−	normal	[16,18,19]
ICF	+	normal	NA	normal	[4,22]
RMT	+	normal	+/−− (improved)	normal	[4,25]
MEP	−	normal	++/− (improved)	normal	[4,25]

## Data Availability

No new data were created or analyzed in this study. Data sharing is not applicable to this article.

## Abbreviations

TMS	transcranial magnetic stimulation
MT	motor threshold
MEP	motor-evoked potential
SICI	short-interval intracortical inhibition
ICF	intracortical facilitation
ppTMS	paired-pulse transcranial magnetic stimulation
AH	affected hemisphere
UH	unaffected hemisphere
M1	primary motor cortex
LTP	long-term potentiation
LTD	long-term depression
ISI	inter-stimulus interval
GABAA	gamma-aminobutyric acid type A
ICI	intracortical inhibition
NMDA	N-methyl-D-aspartate
iSP	ipsilateral silent period
IHI	interhemispheric inhibition
rTMS	repetitive transcranial magnetic stimulation
TBS	theta burst stimulation
